# High‐throughput sequencing approach in analysis of microbial communities colonizing natural gas pipelines

**DOI:** 10.1002/mbo3.806

**Published:** 2019-02-06

**Authors:** Agnieszka Staniszewska, Alina Kunicka‐Styczyńska, Anna Otlewska, Jan Gawor, Robert Gromadka, Karolina Żuchniewicz, Krzysztof Ziemiński

**Affiliations:** ^1^ Faculty of Biotechnology and Food Sciences Institute of Fermentation Technology and Microbiology Lodz University of Technology Lodz Poland; ^2^ DNA Sequencing and Oligonucleotide Synthesis Laboratory Institute of Biochemistry and Biophysics Polish Academy of Science Warsaw Poland

**Keywords:** high‐throughput sequencing, microbial communities, microbiomes, natural gas pipelines

## Abstract

This study provides a deep modern insight into the phylogenetic diversity among bacterial consortia found in working and nonworking high‐methane natural gas pipelines located in Poland. The working pipeline was characterized by lower biodiversity (140–154 bacterial genera from 22 to 23 classes, depending on the source of the debris) in comparison to the off‐gas pipeline (169 bacterial genera from 23 classes). The sediment recovered from the working pipeline contained mostly DNA identified as belonging to the phylum *Firmicutes* (66.4%–45.9% operational taxonomic units [OTUs]), predominantly *Bacillus* (41.4%–31.1% OTUs) followed by *Lysinibacillus* (2.6%–1.5% OTUs) and *Clostridium* (2.4%–1.8% OTUs). In the nonworking pipeline, *Proteobacteria* (46.8% OTUs) and *Cyanobacteria* (27.8% OTUs) were dominant. Over 30% of the *Proteobacteria* sequences showed homologies to *Gammaproteobacteria*, with *Pseudomonas* (7.1%), *Enhydrobacter* (2.1%), *Stenotrophomonas* (0.5%), and *Haempohilus* (0.4%) among the others. Differences were noted in terms of the chemical compositions of deposits originating from the working and nonworking gas pipelines. The deposits from the nonworking gas pipeline contained iron, as well as carbon (42.58%), sulphur (15.27%), and oxygen (15.32%). This composition can be linked to both the quantity and type of the resident microorganisms. The presence of a considerable amount of silicon (17.42%), and of aluminum, potassium, calcium, and magnesium at detectable levels, may likewise affect the metabolic activity of the resident consortia in the working gas pipeline. All the analyzed sediments included both bacteria known for causing and intensifying corrosion (e.g., *Pseudomonas*,* Desulfovibrio*,* Shewanella*,* Serratia*) and bacteria that can protect the surface of pipelines against deterioration (e.g., *Bacillus*). Biocorrosion is not related to a single mechanism or one species of microorganism, but results from the multidirectional activity of multiple microbial communities. The analysis presented here of the state of the microbiome in a gas pipeline during the real gas transport is a particularly valuable element of this work.

## 1 INTRODUCTION

According to the estimates, about 20% of global natural gas deposits originate from biogenic processes (Rice & Claypool, 1981). The creation of these deposits is attributed to microorganisms, mainly methanogenic archaea and sulphate‐reducing bacteria (Katayama et al., 2015; Turkiewicz, 2009). However, studies of microbiomes in natural gas wells have found wide microbial diversity, especially in the case of unconventional gas resources (Davis, Struchtemeyer, & Elshahed, 2012; Katayama et al., 2015; Mochimaru et al., 2007; Yoshioka, Mochimaru, Sakata, Takeda, & Yoshida, 2015). Some microorganisms found in underground gas storage (UGS) reservoirs and caverns may have been transferred along with the transported fuel (Ivanova, Borzenkov, Tarasov, Milekhina, & Belyaev, 2007a,b). Autochthonous microflora, including methanogens such as *Methanobacterium* and acetogenic *Eubacterium*, is common in gas pipeline networks, but also microorganisms belonging to the phyla *Acidobacteria*,* Actinobacteria*,* Bacterioidetes, Cyanobacteria*,* Firmicutes*,* Gammatimonadetes*,* Planctomycetes*,* Proteobacteria,* and *Verrucomicrobia* that usually inhabit drilling mud and fracturing fluids (Struchtemeyer, Davis, & Elshahed, 2011). Microorganisms can also be introduced into gas networks during pipeline replacements, repairs, or extensions. In such cases, the gas lines become contaminated by microbes that usually inhabit soil, water, and air ecosystems and which are not specific to natural gas—that is, *Bacillus*,* Clostridium*,* Pseudomonas*.

Natural gas consists mainly of methane, which determines its heating value. However, it also includes impurities, such as sulfur compounds, solid particles, and water vapor below its dew point. For transportation and usage, natural gas is purified to meet differing standards for various countries. For example, the maximum water content acceptable in “dry” gas ranges from 84 ppm (Canada and Europe) to 147 ppm (the Middle East and Southern USA) (El‐Sherik, 2016). However, there are no standards concerning the microbial purity of transported natural gas.

The development of microorganisms in natural gas pipelines may be promoted by certain compounds of the pipe. Steel, consisting mainly of iron and a carbon admixture, as well as small amounts of sulphur and phosphorus, promotes colonization of the inner surfaces of pipes. Damage due to the metabolic activity of microorganisms is referred to as Microorganisms Induced Corrosion or biodeterioration. It results in the development of biofilms and the formation of “black powder” deposits. It has been estimated that approximately 40% of all corrosion cases in industry are of biological origin (Jan‐Roblero et al., 2008; Rajasekar, Anandkumar, Maruthamuthu, Ting, & Rahman, 2010). Damage due to biological corrosion is responsible for up to 20%–30% of total costs of service, predominantly because of the corrosion of gas pipelines (AlAbbas et al., 2012).

No previous study of the microbiome in gas pipelines has been conducted in Poland. The identification of microbial consortia in these ecological niches may be associated with the corrosion of natural gas transmission lines. The aim of our study was to investigate the phylogenetic diversity of bacterial environments in working and nonworking natural gas pipelines located in Poland. Furthermore, the composition of the microbiomes was discussed in the aspects of internal chemical changes to the surface of the pipelines.

## 2 EXPERIMENTAL PROCEDURES

### 2.1 Sampling

Four samples of sediments were collected from the inner surfaces of two DN 350 natural gas pipelines in Poland: M1—Piotrków Trybunalski, the Warta River; M2—Piotrków Trybunalski, Sworzyce. Sediments were scraped from a surface area of 25 cm^2^ and placed in sterile plastic vessels. The samples were collected during the dismantlement of the M1 transmission line and the overhaul of the M2 gas pipeline by the network operator. The deposits from the nonworking gas pipeline (sample M1‐1) and the working pipeline (samples M2‐1, M2‐2, and M2‐3) were subjected to metagenomic and scanning electron microscopy/energy dispersive X‐ray spectroscopy (SEM/EDS) analysis. Fragments from the carbon steel gas pipelines on both the M1 and M2 lines were collected and their chemical compositions submitted to SEM/EDS analysis.

### 2.2 Natural gas pipeline characteristics

The gas pipelines had been protected against external factors by a bituminous coating and cathodic protection. The gas lines transported high‐methane gas with a maximum working pressure of 3.2 MPa, from domestic deposits in the Podkarpacie region. Detailed descriptions of the gas pipelines and collected samples are given in Table 1.

**Table 1:** Description of gas pipelines and collected samples

Deposit sample	Sampling	Gas pipeline	Construction year	Gas pipeline status
M1‐1	Average^a^	M1—Piotrków Trybunalski—the Warta River (gas route direction N‐S)	1958	Nonworking (closed from 1 year)
M2‐1	Bottom part	M2—Piotrków Trybunalski—Sworzyce (gas route direction NW‐SE)	1975	Working
M2‐3	Upper part			
M2‐2	Dewaterer			

a Average sample originated from different parts of the pipeline.

### 2.3 SEM/EDS microstructure analysis

The surfaces of the pipeline and their chemical compositions were analyzed using X‐ray microanalysis. The microstructure was examined using an S‐3000N SEM (Hitachi, Japan) equipped with an EDS microanalyzer (Thermo NORAN, Madison), according to the method described by Burnat, Walkowiak‐Przybyło, Błaszczyk, and Klimek (2013) and Pietnicki, Wołowiec, and Klimek (2014). Tests were carried out on uncoated samples, prepared in the form of fracture surfaces. The results were presented as the percentage of individual elements in the surface layer of the carbon steel (the average values of the elemental composition in % wt).

### 2.4 DNA extraction

Total genomic DNA was isolated from 250 mg samples using a Soil DNA Extraction Kit (Eurx, Poland), following the manufacturer's instructions. The samples were first pulverized using a TissueLyser apparatus (Qiagen, Germany) with 0.4–0.6 diameter glass beads (Sartorius AG, Germany). After isolation, the quality of the DNA was analyzed by running the sample on 1% (w/v) agarose gel. Template quantity was measured by fluorimetry using Qubit 2.0 and High Sensitivity Picogreen reagents (Invitrogen/Life Technologies). Amplification of the conserved bacterial 16SrRNA gene fragment covering V3 and V4 regions was performed in triplicate with the use of the gene universal specific primers 341F and 785R (Klindworth et al., 2012).

### 2.5 PCR amplification and sequencing

The obtained amplicons, *c*. 450 bp in size, were analyzed in 1% (w/v) agarose gel and purified by AMPure XP magnetic beads (Beckman Coulter, Inc.). The amplicon libraries were pooled in an equimolar ratio and indexed according to the Nextera indexing strategy by PCR (Illumina, Inc., San Diego, CA). Sample indexing enabled pooling of the amplicons for sequencing and further extraction of the sample sequence reads, from the large batch of sequencing data. The 16S amplicons were sequenced on an MiSeq sequencer in the DNA Sequencing and Oligonuleotide Synthesis Laboratory IBB PAS (Warsaw, Poland) in paired end mode using a 600 cycle v3 chemistry kit (Illumina, Inc., San Diego, CA).

### 2.6 Sequencing data analysis

The sequence reads were filtered by quality using a FastX toolkit and processed using a QIIME pipeline (http://qiime.org/). Paired reads were merged into contigs. Sequences were grouped based on their taxonomic classification and highly similar sequences were clustered into operational taxonomic units (OTUs). Finally, the obtained data were aligned against a curated 16S (Ribosomal Database Project) database. Raw data files in the FASTQ format were deposited in the NCBI Sequence Read Archive (SRA) under the study accession number SUB 3239483 with the Bioproject number PRJNA431861. Phylogenetic trees were constructed using MEGAN software (Huson et al., 2016).

## 3 RESULTS AND DISCUSSION

**Figure 1:**
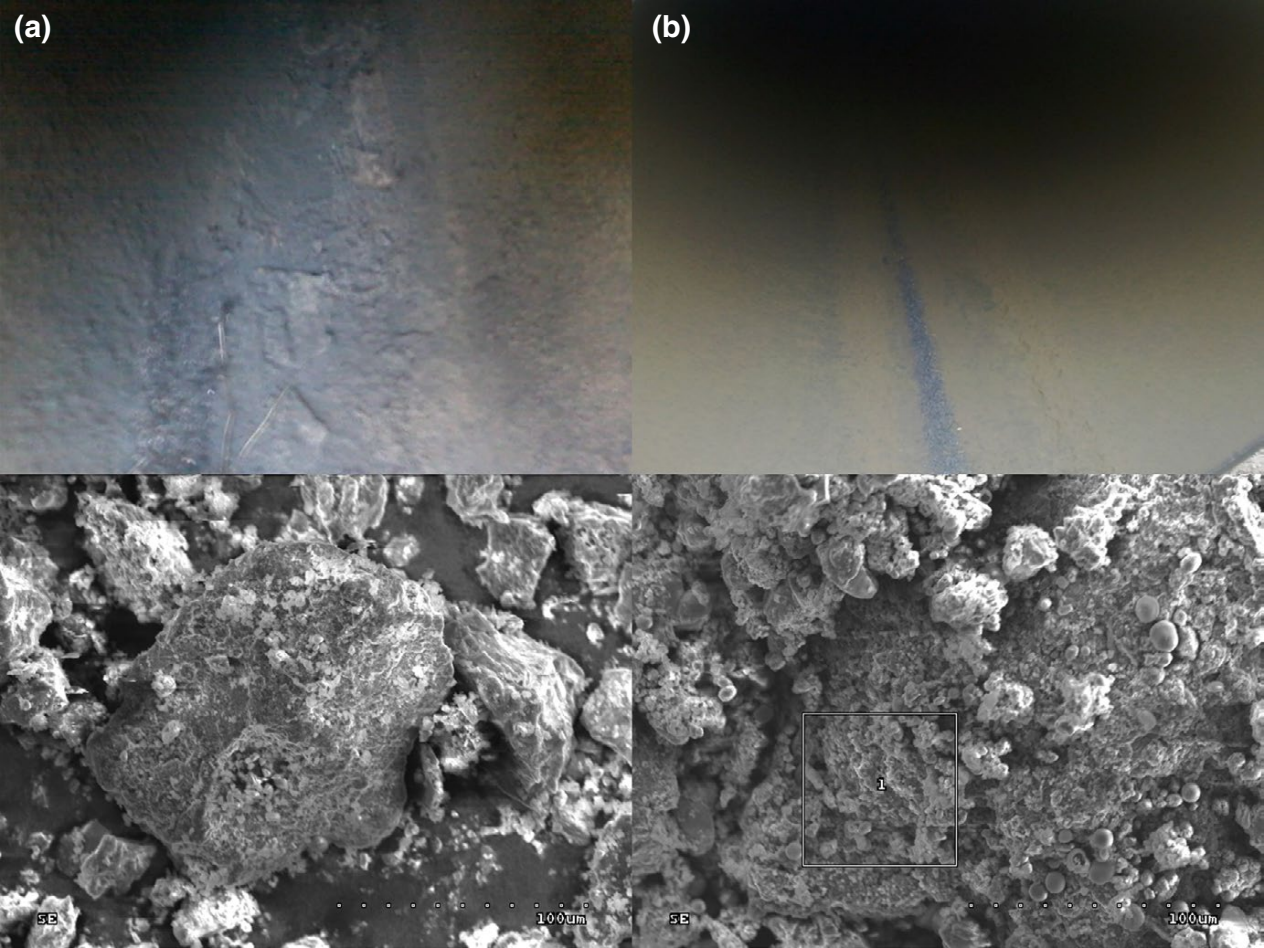
The inner surface of DN 350 natural gas pipelines in Poland; (a) M1—Piotrków Trybunalski—the Warta River and (b) M2—Piotrków Trybunalski—Sworzyce and beneath the scanning electron microscopy images of their corresponding deposits (S‐3000N scanning electron microscope; Hitachi, Japan)

**Figure 2:**
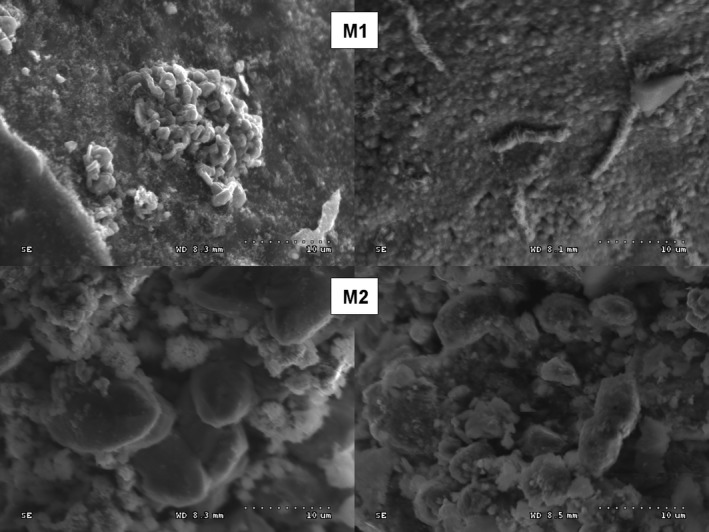
Cells of microorganisms on the inner surface of DN 350 natural gas pipelines in Poland; M1—Piotrków Trybunalski—the Warta River; M2—Piotrków Trybunalski—Sworzyce (S‐3000N scanning electron microscope; Hitachi, Japan)

**Figure 3:**
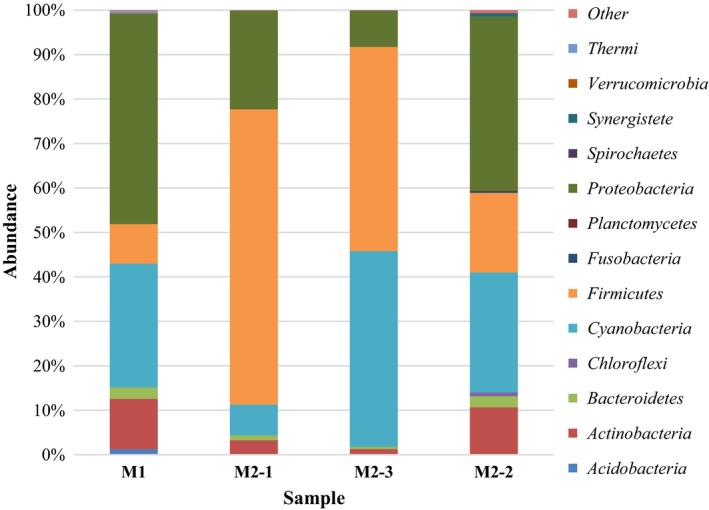
Percentage share of each bacterial phylum making up the total number of identified strains colonizing natural gas pipelines

**Figure 4:**
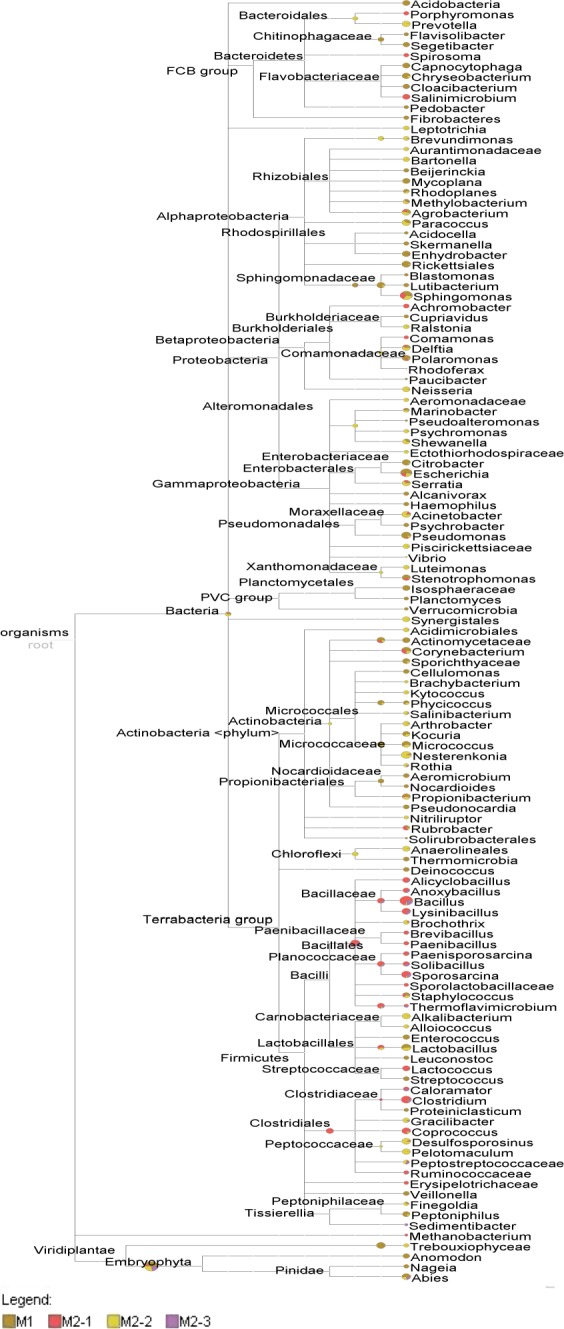
Neighbor‐joining phylogenetic tree constructed on bacterial 16S rRNA gene sequences retrieved from high‐throughput sequencing of gas industry pipeline samples. Numbers at nodes represent the percentages of occurrence of nodes in 1,000 bootstrap trials. Only bootstrap values >50% are indicated. The scale bar represents the expected number of substitutions per nucleotide position

Two natural gas pipelines, one nonworking (M1) and another working (M2), were investigated in this study. Natural gas pipelines are an extreme habitat for microorganisms, in which multidirectional stress factors cause the selection of microbial communities. The inner surfaces of the pipelines were covered by a nonhomogeneous layer of a sediment (Figure 1). SEM images of the surfaces of collected debris revealed the presence of microbial cells (Figure 2). DNA high‐throughput sequencing data showed the prevalence of 169 genera of bacteria, belonging to 23 different classes in a sediment sample (M1‐1) collected from the nonworking gas pipeline (Figure 3). Sequences of *Proteobacteria* and *Cyanobacteria* were dominant in this sample and constituted 46.8% and 27.8% of the OTU, respectively (Figure 4). Over 30% of the *Proteobacteria* sequences showed a homology to *Gammaproteobacteria*. In this group, *Pseudomonas* (7.1%), *Enhydrobacter* (2.1%), *Stenotrophomonas* (0.5%), and *Haempohilus* (0.4%) were identified. *Pseudomonas* is classified as metal‐reducing bacteria, which causes corrosion by dissolving passive sediments on the surface of the metal, or by converting them to a less stable reduced form. It has the ability to reduce iron and manganese oxides, at a reaction rate that depends on the types of deposit on the surface of the pipe (Beech & Gaylarde, 1999; Nawrocki & Świetlik, 2011). Like *Pseudomonas* sp., *Shewanella* sp. (i.e., *Shewanella putrefaciens*) can contribute to biocorrosion, by reducing solid iron oxides (III) to Fe^2+^, causing corrosion deposits on the surface to crumble (Jan‐Roblero, Romero, Amaya, & Le Borgne, 2004; Zhu, Lubeck, & Kibane, 2003).

Bacteria from the *Enterobacteriaceae* family accounted for 18.6% of OTUs in sample M1‐1, while the percentage of particular types did not exceed 0.2% (*Citrobacter*,* Enterobacter*,* Erwinia*,* Serratia,* and *Shigella*) (Figure 3). Several members of the *Enterobacteriaceae* family (*Citrobacter*,* Enterobacter*,* Klebsiella*,* Proteus,* and *Serratia*) have been detected in biofilms collected from corroded seawater, oil, and gas pipelines (Bermont‐Bouis, Janvier, Grimont, Dupont, & Vallaeys, 2007; Jan‐Roblero et al., 2004, 2008; Neria‐González, Wang, Ramírez, Romero, & Hernández‐Rodríguez, 2006; Rajasekar et al., 2007). *Enterobacter* (e.g., *E. agglomerans*) can grow by coupling the reduction in Fe (III), Mn (IV), or Cr (VI) by the oxidation of acetate and H_2_ (Jan‐Roblero et al., 2004). Another member of the *Enterobacteriaceae* family, *Citrobacter* sp., is able to accumulate metals on the surface of its cells and binds heavy metals by enzymatic metal‐phosphate deposition. Moreover, some species of *Citrobacter* (e.g., *C. freundii* and *C. amalonaticus*) can produce hydrogen sulfide and cause steel corrosion, which is difficult to detect (Angeles‐Chavez, Romero, Amaya, Martinez, & Perez, 2001; Kodaka, Mizuochi, Honda, & Yamaguchi, 2000). *Actinobacteria* and *Firmicutes* composed, respectively, 11.3% and 8.9% of the total OTUs in sample M1‐1, while the percentage of other classes did not exceed 3% (*Bacteroidetes*—2.6%, *Acidobacteria*—1.3%, *Thermi*—0.4%, *Verrucomicrobia*—0.1%) (Figure 3).

The sediments from the bottom (M2‐1) and upper (M2‐3) of the working pipeline transmitting high‐methane natural gas were characterized by lower microbial diversity in comparison to the sediment in the off‐gas pipeline. In total, 140 and 154 bacterial genera from 23 and 22 classes, respectively, were identified (Figure 4). The lower microbial diversity of the deposits in the operating gas pipeline may be related to the shortage of nutrients and water content (Turkiewicz, 2011). The composition of the transmitted gas, as well as the presence of chemical components in the steel pipes (sulphur, phosphorus, iron, manganese) may also promote the development of specialized groups of microorganisms, which are capable of growth and proliferation under extreme environmental conditions.

The DNA recovered from the sediments M2‐1 and M2‐3 belonged mostly to *Firmicutes* (66.4% and 45.9%, respectively), with a predominance of spore‐forming bacteria belonging to *Bacillus* genera (41.4% and 31.1%), followed by *Lysinibacillus* (2.6% and 1.5%) and *Clostridium* (2.4% and 1.8%) (Figure 3). According to Beech and Gaylarde (1999), a significant number of corrosion processes can be attributed in part to slime‐producing bacteria, namely *Bacillus*,* Clostridium*,* Pseudomonas*,* Flavobacterium*, as well as to the sulfate‐reducing bacteria (SRB), *Desulfovibrio* and *Desulfotomaculum*. Extracellular polymeric substances (EPS) produced during the growth of biofilms facilitate irreversible attachment of microbial cells to the pipeline surface, to inorganic precipitates derived from the bulk aqueous phase and/or to corrosion products from the metal substratum. A study by Scotto, Beggiato, Marcenaro, and Dellepiane (1993) showed that only 10 ng/cm^2^ of EPS can initiate corrosion of stainless steel.

Generally, *Clostridium* do not reduce sulfate by a dissimilatory pathway, although some species are able to reduce sulfate or thiosulfate and accumulate sulphides—a corrosive agent to steel and other materials (Jan‐Roblero et al., 2008). It is also possible that corrosion processes may be initiated or accelerated by the coexistence in gas pipelines and synergistic relationship between aerobic strains of *Bacillus* and strictly anaerobic *Clostridium*. *Bacillus* also contributes to the formation of a polysaccharide matrix during biofilm formation on the internal surfaces of pipelines, simultaneously consuming oxygen and producing primary metabolites. As a result, anaerobic conditions are created that promote the development of both *Clostridium* and SRB. In another respect, however, EPS may protect the surface of pipelines against corrosion processes. It has been demonstrated that a thermophilic consortium of *Bacillus* and *Deleya marina* produces polysaccharides that bind metal particles, reducing the degree of carbon steel corrosion by up to 94% (Beech & Gaylarde, 1999). In addition to creating a protective EPS or stabilizing a pre‐existing biofilm on the surface of metal, microorganisms can also contribute to inhibit corrosion by neutralizing the action of corrosive substances and decreasing the cathodic rate, by consuming oxygen thought respiratory activity (Kip & van Veen, 2015; Videla & Herrera, 2005).

SRB belonging to *Desulfosporosinus* and *Desulfotomaculum* (*Firmicutes*) as well as *Desulfovibrio* (*Proteobacteria*) were found in the investigated samples. *Desulfosporosinus* was the most abundant of all the SRBs identified, as it was demonstrated in the phylogenetic tree (Figure 4). SRB occurred the most frequently in the working gas pipeline (M2). Bacteria belonging to *Desulfosporosinus* were identified in all sediment samples from the working gas pipeline: bottom (M2‐1), top (M2‐3), and in the dewaterer (M2‐2), constituting 0.26%, 0.18%, and 1.63% of OTUs, respectively. SRB belonging to *Desulfotomaculum* were detected only in the dewaterer (M2‐2) and accounted for <0.003% of OTUs. In the sediment sample collected from the nonworking gas pipeline (M1), of all the identified SRBs only *Desulfovibrio* was detected, at 0.003% of OTUs. According to Jan‐Roblero et al. (2004), even if SRBs are present in only small quantities, they may significantly contribute to corrosion. Both *Desulfotomaculum* and *Desulfovibrio* have been previously reported as being associated with microbial communities in gas industry pipelines (in Poland, USA and Mexico), and with sites of microbially influenced corrosion (Jan‐Roblero et al., 2004, 2008; Zhu et al., 2003). However, there is no indication in the literature linking *Desulfosporosinus* with the formation of corrosive biofilm and, to the best knowledge of the authors, this is the first study to report the detection of this species in a gas pipeline. To date, these bacteria have been noted only in oil‐polluted or petroleum‐contaminated sediments (Allen et al., 2007).

In the sample of sediment taken from the bottom of the gas pipeline (M2‐1), the presence of anaerobic acetogenic bacteria *Sporomusa* was identified. These bacteria are gram negative and also express the ability to form endospores. The occurrence of both these features in one microorganism is extremely rare. *Sporomusa* (mainly *S. ovata*) was isolated from an UGS reservoir in Russia (Balk et al., 2010). The *Sporomusa* isolates were found to be capable of perchlorate reduction. According to Drake, Küsel, and Matthies (2006), *Sporomusa* bacteria derive energy by CO_2_ reduction in acetate via acetogenesis. Their acetogens utilize the acetyl‐CoA pathway for the energy‐conserving CO_2_‐fixing process (Drake, 2009). As with the M1‐1 sediment, in the M2‐1 and M2‐3 samples significant percentages were found of *Cyanobacteria* (27.8% and 44.0%) and *Proteobacteria* (22.2% and 8.1%). *Sphingomonas* were dominant among *Proteobacteria*, representing 9.7% and 1.6% of the total bacterial pool in M2‐1 and M2‐3, respectively. From this class of bacteria, poly(3‐hydroxybutyrate‐co‐3‐hydroxyvalerate)‐degrading denitrifiers *Comamonas* sp., previously observed (*Comamonas denitrificans*) by Zhu et al. (2003) in microbial communities collected from different three gas pipelines, were also detected.

In gas pipelines, a dewaterer is used to collect condensate water released from gas and solid contaminants. The dewaterer is installed at the lowest point in the high pressure line, to ensure the proper operation. Due to its structure and functions, the dewaterer in the analyzed pipeline was found to serve as a specific ecological niche for bacterial growth. Sample M2‐2 from sediment collected from the dewaterer was slightly different from the other samples in terms of the microbial species it contained. As in the case of sample M1‐1, the largest percentage of the analyzed sequences was recorded for the *Proteobacteria* class (39%), among which bacteria from the genera *Sphingomonas* (10%), *Pseudomonas* (1.9%), and *Delftia* (1.4%), characteristic for water environments, were detected. *Actinobacteria* accounted for more than 10% of the total OTUs in this sample, of which *Nesterenkonia* (1.6%), *Propionibacterium* (1.3%), *Corynebacterium* (1.2%), *Arthrobacter* and *Micrococcus* (0.8%) were the most abundant. Of the listed microorganisms, only *Propionibacterium* had been detected previously, by Zhu et al. (2003) in gas pipelines in USA.

The environment in natural gas pipelines is formed by the components of the transmitted gas and the carbon steel constituents of the gas pipelines, as well as by the metabolites of the inhabiting microorganisms. In the atmosphere of the analyzed natural gas grid, the main components were hydrocarbons, which can be a source of energy for microorganisms. According to a report by Polska Spółka Gazownictwa Sp. z o. o., the composition (in molar %) of high‐methane natural gas sent through the examined gas pipelines (from the direction of Podkarpacie) was as follows: methane—95.104 ± 0.062, ethane—2.409 ± 0.041, propane—0.631 ± 0.013, nitrogen—1.024 ± 0.002, CO_2_—0.564 ± 0.007, I‐pentane—0.027 ± 0.0003, N‐pentane—0.018 ± 0.0002, I‐butane—0.090 ± 0.002, N‐butane—0.100 ± 0.002. In both gas pipelines, M1 and M2, the amount of possible nutrients available to the organisms with natural gas, which is reflected by the similar composition of dominant microorganisms.

The macro‐ and microelements of steel also affect the environmental conditions and the microbiome in gas pipelines. The chemical compositions of the pipelines are presented in Table 2. The most striking differences between the tested pipelines concern the phosphorus and the sulphur contents. Even small differences in the compositions of steel pipelines can affect the diversity of microorganisms. Therefore, the 4‐ and 6‐times lower content of P and S in the composition of the M2 steel pipeline may have contributed to the lower microbial diversity in samples from its deposit. The simultaneous decrease in the number of biogenic elements and lower bacterial diversity creates favorable conditions for the development of Archaea.

**Table 2:** Elementary composition of carbon steel of the gas pipelines and the deposits formed on the gas pipelines internal surface (% by weight)

Compounds	Gas pipeline M1		Gas pipeline M2	
	Carbon steel	Deposits	Carbon steel	Deposits
Fe	99.04	35.35	99.29	38.59
C	0.11	42.58	0.10	nd
Mn	0.46	nd	0.48	nd
Si	0.19	nd	0.10	17.42
P	0.06	nd	0.01	nd
S	0.04	15.27	0.01	nd
Cu	0.10	nd	nd	nd
O	nd	15.32	nd	35.01
Al	nd	nd	nd	5.56
K	nd	nd	nd	1.55
Ca	nd	nd	nd	1.34
Mg	nd	nd	nd	0.52

*Note*

nd: not detected.

It is interesting to note the differences between the chemical compositions of deposit samples from the working and nonworking gas pipelines. The deposits from the nonworking gas pipeline (M1) were composed of iron, as well as carbon (42.58%), sulphur (15.27%), and oxygen (15.32%). The presence of these compounds may be related both to the quantity and type of the resident microorganisms. The nonworking gas pipeline, filled with air, was also a convenient environment for the development of aerobes and semi‐anaerobes. The presence of considerable amounts of silicon (17.42%), and of detectable levels of aluminum, potassium, calcium, and magnesium, can attribute to the metabolic activity of the residentiary consortium in the working gas pipeline (M2) (Table 2). Impurities carried with the flowing gas may also have been a source of the microelements detected in these sediments. The compounds detected in the samples are also described as the elements of the “black powder” created on the inner surfaces of the gas pipes. “Black powder” is composed mostly of iron oxides, iron hydroxides and siderite, and contains impurities such as elemental sulphur, hydrocarbons, metal fragments, sand, and dust. “Black Powder” is also considered as corrosive to gas pipeline materials (AlAbbas et al., 2012; Staniszewska, Kunicka‐Styczyńska, & Ziemiński, 2017). Double the amount of oxygen was found in the sediment from the M‐2 gas pipeline compared to that from the non‐working pipeline. This may be the result of activity by *Cyanobacteria*, especially in the upper part of this pipeline (sample M2‐3) (Figure 3). *Proteobacteria* in the nonworking gas pipeline (M1) and in the dewaterer (M2‐2) may also be linked to oxygenation.

## 4 CONCLUSIONS

This study has presented an in‐depth analysis of the microbial diversity in consortia residing in natural gas transmission lines in Poland. In the currently available literature describing microorganisms in gas pipelines around the world, this is the first analysis of the microbial community in the Polish gas network, providing a valuable point of comparison for understanding how microbial consortia spread, and affect the pipelines they colonize. The study was conducted in an operating gas network. This presentation of the current state of the microbiome in gas pipelines during gas transport is a particularly valuable component in this work.

The gas pipelines were found to be a more favorable environment for the growth and development of bacteria than for Archaea. Between 140 (M2‐1) and 169 (M1‐1) different genera of bacteria were identified in samples from the pipelines, and only one Archaea, *Methanobacterium* (M2‐1). The presence of Archaea, which is characteristic of the microflora in natural gas deposits (Ivanova et al., 2007a,b), indicates the feasible ability of these microorganisms to be transported with the natural gas in the pipeline gas grid. The following bacteria, also found in a natural UGS facility (Balk, van Gelder, Weelink, & Strams, 2008; Balk et al., 2010; Sobodkina et al., 2013), were identified: *Desulfovibrio* (M1‐1, M2‐2 and M2‐3 samples), *Sporomusa* (M2‐1 and M2‐3 samples), *Desulfotomaculum* (M2‐2 sample). The occurrence of these bacteria in the analyzed microbiomes supports the hypothesis that microorganisms are capable of spreading through the natural gas grid with the transported fuel. A group of microflora autochthonous to the Polish gas grid could not be identified.

Elements in the gas network devoid of fresh gas portions (M1‐1) and purged of direct gas flow (M2‐2) differed in terms of the number and type of microorganisms from the environments with a constant gas supply (M2‐1 and M2‐3). Variations in the composition of the steel used in the construction of gas pipelines may account in some measure for the microbial diversity of the analyzed consortia. All the analyzed sediments included both bacteria known for causing and intensifying corrosion (including: *Pseudomonas*,* Desulfovibrio*,* Shewanella*,* Serratia*) and bacteria that can protect the surface of pipelines against deterioration (e.g., *Bacillus*). The environments differed in terms of the abundance of these groups. For example, in the M1‐1 sample *Pseudomonas* (7.1% OTUs) predominated, while *Bacillus* constituted only 1.1% of OTUs. In the M2‐1 sample, in contrast, *Bacillus* constituted the dominant group (41.4% of OTUs) while *Pseudomonas* was only <0.2% OTUs. Given such variations in the data, predicting the rate of biocorrosion is difficult. However, the mechanisms for initiating or inhibiting biocorrosion are usually associated with changes in the environmental parameters at the metal‐solution interface, due to microbial activity (Videla & Herrera, 2005). It should also be emphasized that biocorrosion is not related to a single mechanism or the occurrence of one species of microorganism, but most often results from the multidirectional activity of multiple microbial species. Therefore, estimating the progress of biocorrosion on the basis of data regarding the diversity and abundance of individual microorganisms is not straightforward, and requires further research.

## CONFLICT OF INTEREST

No conflict of interests is declared.

## AUTHORS CONTRIBUTION

A.S. involved in experiment design and carried out experiments, collected methodology and experimental data, deposited sequences in the NCBI database, preliminary preparation of figures, manuscript preparation; A.K.S. provided guidance on experiment design, involved in preparation of figures, manuscript preparation, manuscript revision, corresponding author; A.O. assisted in experimental design, involved in preparation of figures, manuscript preparation; J.G., R.G., K.Ż. performed all metagenomics processing, deposited sequences in the NCBI database, phylogenetic tree preparation; K.Z. involved in experiment design and manuscript preparation.

## ETHICS STATEMENT

None required.

## Data Availability

Raw sequence data have been deposited to NCBI SRA under the Bioproject number PRJNA431861.
